# Models Analyses for Allelopathic Effects of Chicory at Equivalent Coupling of Nitrogen Supply and pH Level on *F. arundinacea*, *T. repens* and *M. sativa*

**DOI:** 10.1371/journal.pone.0031670

**Published:** 2012-02-22

**Authors:** Quanzhen Wang, Bao Xie, Chunhui Wu, Guo Chen, Zhengwei Wang, Jian Cui, Tianming Hu, Pawel Wiatrak

**Affiliations:** 1 Department of Grassland Science, College of Animal Sciences and Technology, Northwest Agriculture and Forestry University, Yangling, Shaanxi Province, People's Republic of China; 2 Department of Plant Science, College of life Science, Northwest Agriculture and Forestry University, Yangling, Shaanxi Province, People's Republic of China; 3 Clemson University/Edisto Research and Education Center, Blackville, South Carolina, United States of America; Cairo University, Egypt

## Abstract

Alllelopathic potential of chicory was investigated by evaluating its effect on seed germination, soluble sugar, malondialdehyde (MDA) and the chlorophyll content of three target plants species (*Festuca arundinacea*, *Trifolium repens* and *Medicago sativa*). The secretion of allelochemicals was regulated by keeping the donor plant (chicory) separate from the three target plant species and using different pH and nitrogen levels. Leachates from donor pots with different pH levels and nitrogen concentrations continuously irrigated the target pots containing the seedlings. The allelopathic effects of the chicory at equivalent coupling of nitrogen supply and pH level on the three target plants species were explored via models analyses. The results suggested a positive effect of nitrogen supply and pH level on allelochemical secretion from chicory plants. The nitrogen supply and pH level were located at a rectangular area defined by 149 to 168 mg/l nitrogen supply combining 4.95 to 7.0 pH value and point located at nitrogen supply 177 mg/l, pH 6.33 when they were in equivalent coupling effects; whereas the inhibitory effects of equivalent coupling nitrogen supply and pH level were located at rectangular area defined by 125 to 131 mg/l nitrogen supply combining 6.71 to 6.88 pH value and two points respectively located at nitrogen supply 180 mg/l with pH 6.38 and nitrogen supply 166 mg/l with pH 7.59. Aqueous extracts of chicory fleshy roots and leaves accompanied by treatment at different sand pH values and nitrogen concentrations influenced germination, seedling growth, soluble sugar, MDA and chlorophyll of *F. arundinacea*, *T. repens* and *M. sativa*. Additionally, we determined the phenolics contents of root and leaf aqueous extracts, which were 0.104% and 0.044% on average, respectively.

## Introduction

Allelopathy was first defined by Molisch as a direct or indirect interaction among plants that causes harmful or beneficial effects through the release of chemicals [1]–[3]. A number of scientists have recognized the importance of allelopathy and other molecular mechanisms in crop and forage production. Hopefully, the technologies developed as a result of that recognition will significantly reduce the use of herbicides while still effectively protecting biodiversity. In turn, these advances could dramatically increase agricultural production and enhance the quality of many crop products [4]–[8]. Allelopathy plays an important role in agro-ecosystems and often has a great influence on the interactions of biotic communities [9], such as the vegetation community composition [10]. Such influences and interactions are mainly the result of allelochemical release from donor plants to target plants [9]. Allelochemicals are mainly a variety of secondary metabolites which include organic acids, phenols, terpenes, fatty acids and L-tryptophan [11], [12]. Researchers and farmers have both widely proven that these secondly metabolites possess insecticidal, antimicrobial and nematicidal properties. Additionally, microbes can use these secondary metabolites as carbon sources, and bacteria utilize these compounds for quorum-sensing [13]. Thus, allelochemical secretion plays a major role in the success of plants and in maintaining ecological balance.

Chicory (*Cichorium intybus L.*), a perennial ratoon plant of the family Compositae, originated in the Mediterranean, central Asia and northern Africa. Cultivation of this plant has been reported as early as the ancient Roman and Greek eras [14], [15]. Chicory is one of the most promising novel plant candidates among the carbohydrates with a potential for utilization in both food and non-food products [16], [17]. Four thousand years ago, chicory roots were used as a substitute for coffee in ancient Egypt [18], [19]. Chicory has a higher content of sodium, zinc, copper, iron, calcium, magnesium and sulphur compared to ryegrass and lucerne [20], [21]. Thus, chicory supplementation in a grass mixture can supply protein, minerals and vitamins to livestock [22]. Grazing chicory has shown a better growth rate than perennial ryegrass or other grasses [23], [24]. The optimal chicory growth temperature is 17–20°C with an assimilation function abating at more than 20°C. Chicory needs sufficient moisture, light and nutrients throughout the growth process. This plant is highly adaptable to acidic and alkaline soil conditions, but exorbitant acidity inhibits its growth.

The gramineae of *F. arundinacea* grows well in fertile, moist, fine loamy soil that is rich in organic matter with a pH value range of 4.7–8.5. This plant does not withstand high temperatures, drought or trampling. It is resistant to half cloudy conditions and is sensitive to fertilizer. It is a plant suited for warm humid subtropical to temperate regions.

The legume of *T. repens* can adapt to all kinds of soil types and grows well in partially acidic environments. *T. repens* is resistant to trimming and trampling and has a strong regeneration ability. In high shade conditions, it grows poorly. It is highly resistant to diseases, harmful insects and gas pollution.

*M. sativa* is a perennial, highly resistant legume forage crop that has adapted to grow in many climates and soil environments. It thrives in dry, warm and sunny climates with less rainy weather. The optimal temperature is 25 to 30°C. An annual rainfall between 400–800 mm facilitates growth, but rainfall over 1,000 mm is detrimental. This alfalfa species has adapted to growth in neutral and slightly alkaline soil, but not in strongly acidic and alkaline soils. The optimum soil pH value is around 7–8.

However, the allelopathic effect of chickory on other grasses is not well documented. Nitrogen is one of the main nutrients required by plants [25]. Inadequate nitrogen supply will limit plant growth and decrease yield potential [2], [26], [27]. Additionally, soil pH can affect nutrient absorption and plant growth [28], [29]. Therefore, we addressed three main questions in our study: 1) Is the germination and growth of *F. arundinacea*, *T. repens* and *M. sativa* affected by aqueous extracts of the fleshy roots and leaves of chicory grown in sands with different pH and nitrogen levels? 2) Do chicory secreting allelopathic compounds in leachates affect soluble sugar, malondialdehyde (MDA) and chlorophyll contents of the three target plant species? 3) How does the integrative effect of nitrogen and pH coupling via donor plant influence seedling growth of the three target plant species?

## Results

### Effects of chicory leaching on the total soluble sugar, MDA and chlorophyll content of target plants

**Figure 1:**
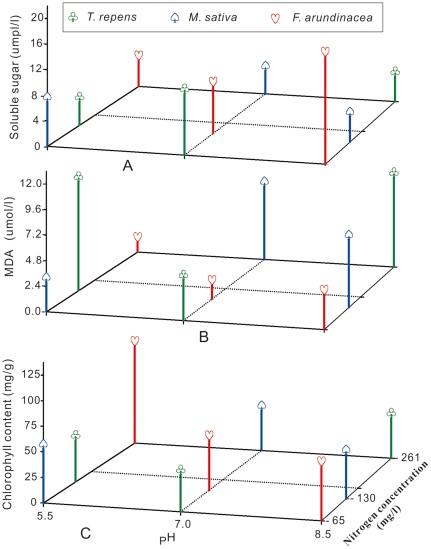
Contents of soluble sugar, MDA and chlorophyll in *T. repens*, *M. sativa* and *F. arundinacea* under orthogonal designed experiment. Note: (A) soluble sugar, (B) MDA and (C) chlorophyll.

The highest soluble sugar content was in *F. arundinacea* under donor condition of nitrogen and pH at 65 mg/l and 8.5 respectively; then comes that in *T. repens* at 65 mg/l of nitrogen and pH = 7.0 (Fig. 1 A). The content of soluble sugar in *F. arundinacea* was higher than that in *T. repens* and *M. sativa* (Fig. 1 A). Both the highest and the second highest MDA content were in *T. repens* under donor condition of 130 mg/l of nitrogen combining pH = 5.5 and 260 mg/l of nitrogen combining pH = 8.5 respectively (Fig. 1 B). Additionally, for *F. arundinacea*, the content of MDA was lower than the other two target plants (Fig. 1 B). The highest chlorophyll content was in *F. arundinacea* under donor condition of 260 mg/l of nitrogen and pH = 5.5 (Fig. 1 C). The variance analysis indicated that the experimental factors, nitrogen pH and target plants, individual effect, the pairwise effects and interacts of the three were significant in terms of the soluble sugar, MDA and chlorophyll content (Table 1).

**Table 1:** The variance analysis of the soluble sugar, MDA and chlorophyll content of the target plants under different treatments of nitrogen and pH.

Source	DF	Pr>F		
		soluble sugar	MDA	Chlorophyll
Nitrogen	2	<.0001	<.0001	0.0015
pH	2	0.0040	0.0005	0.0001
Target plants	2	<.0001	<.0001	<.0001
Nitrogen×pH	4	<.0001	<.0001	<.0001
Nitrogen×Target plants	4	<.0001	<.0001	<.0001
pH×Target plants	4	<.0001	<.0001	<.0001
Nitrogen×pH×Target plants	8	<.0001	<.0001	<.0001
R-Square		0.8259	0.7316	0.8363

### Effects of crude chicory root and leaf water-soluble extract on germination and growth of *T.* repens

**Figure 2:**
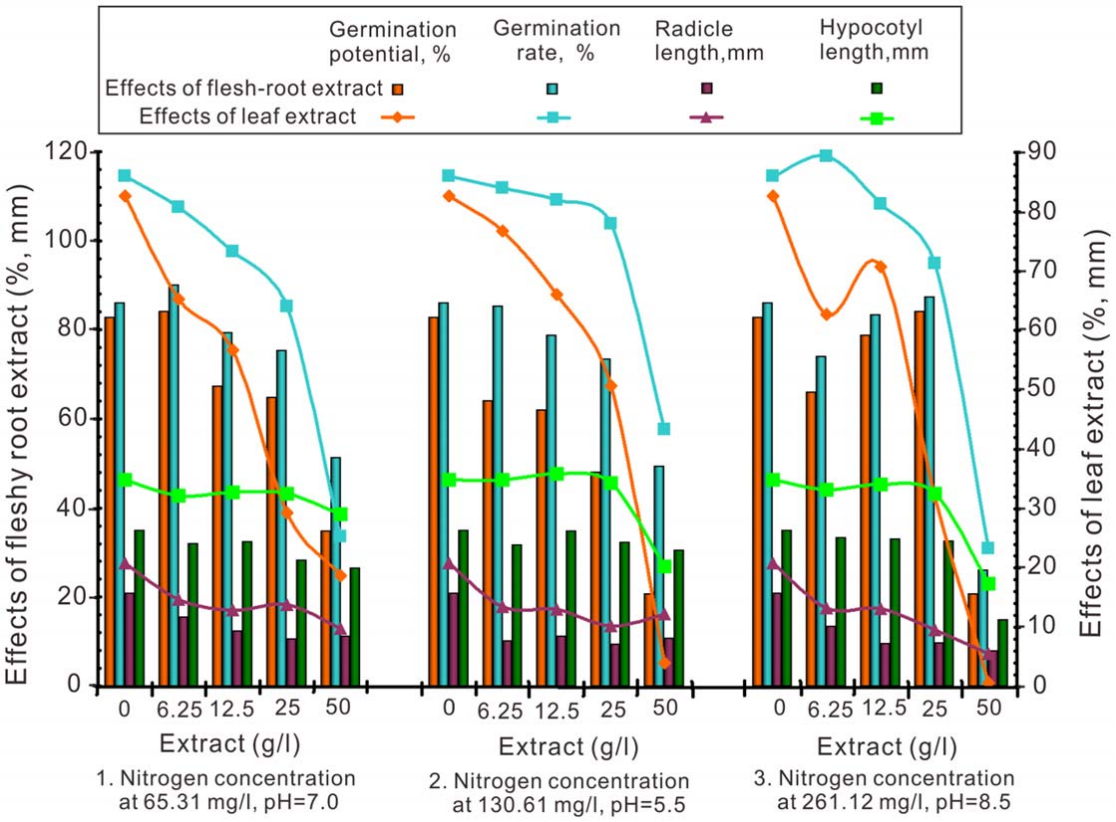
Effects of aquatic lixivium of different concentrations of chicory root and leaf extracts on the germination potential, germination rate, and radicle and hypocotyl length of *T. repens* under varying nitrogen and pH treatments.

The results of the *T. repens* germination potential and rate, radicle and hypocotyl length with the concentrations of chicory root water-soluble extract under three combinations of different pH levels and nitrogen concentrations are shown in Fig. 2. The integrative trends of germination potential, germination rate, lengths of radicle and hypocotyls were decreased with an exception of hypocotyls at lower 25 g/l of extract when the extracts concentration increased from 0 to 50 g/l. The range (R) can described as the degree of influence the factors had on the test results. The effects of the chicory root and leaf extracts at the all pH levels and nitrogen concentration combinations on the germination potential of *T. repens* was as follows: R_3_ (261 mg/L nitrogen at pH 8.5)>R_2_ (130 mg/L nitrogen at pH 5.5)>R_1_ (65 mg/L nitrogen at pH 7.0); and germination rate as follows: R_3_>R_1_>R_2_. The chicory plant produced more secondary metabolites when grown in 261.22 mg/L nitrogen at pH 8.5. Generally, the lower concentrations of chicory root and leaf extracts fail to significantly influence the germination potential and germination rate of *T. repens*, which decreased with an increase in the extract concentration. The 50 g/L treatment significantly inhibited the *T. repens* germination potential and rate.

The ranges of the chicory root extracts from the three pH levels and three nitrogen concentrations combinations on the radicle and hypocotyl length of *T. repens* were as follows: R_3_>R_2_>R_1_ and R_3_>R_1_>R_2_, respectively. In contrast, the range of the chicory leaf extracts from the three pH and three nitrogen concentration combinations on the radicle length of *T. repens* was R_3_>R_1_>R_2_; and on the hypocotyl length was R_3_>R_2_>R_1_. The radicles were more sensitive to inhibition than the hypocotyls. When extract concentrations were very low (6.25 g/L), the growth of the radicle was inhibited. However, the growth of the hypocotyl was normal in lower extract concentrations and was not inhibited until the extract concentration reached 50 g/L or higher.

### Effects of crude chicory leaf and root water-soluble extract on germination and growth of *M. sativa*

**Figure 3:**
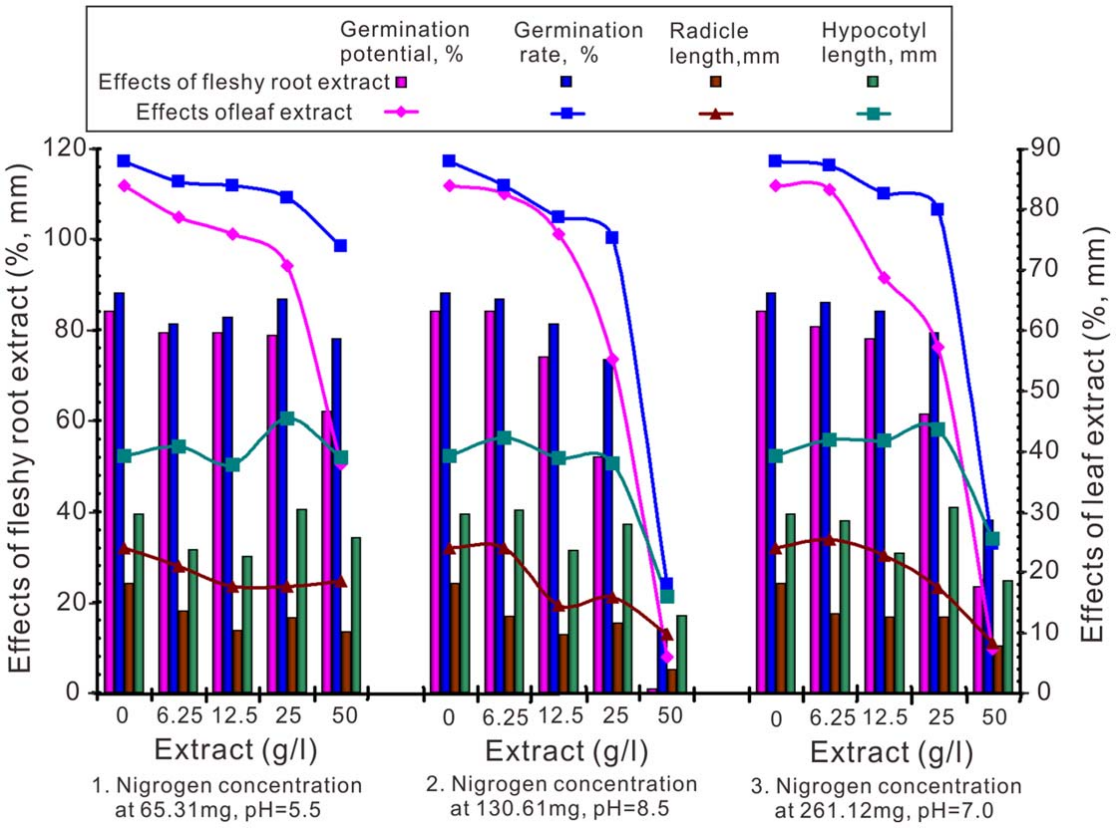
Effects of aquatic lixivium of different concentrations of chicory root and leaf extracts on germination potential, germination rate, and radicle and hypocotyl length of *M. sativa* under varying nitrogen and pH treatments.

The results of the *M. sativa* germination potential and rate, radicle and hypocotyl length with the crude chicory water-soluble extract concentrations under three combinations of both different pH levels and nitrogen concentrations are shown in Fig. 3. The integrative trends of radicle length, germination potential and germination rate at 2 and 3 groups were decreased when the extracts concentration increased from 0 to 50 g/l. Fig. 3 also shows that the extract combination 2 that contains 130.61 mg/L nitrogen with a pH of 8.5 can exhibit a strong inhibiting result especially with the 50 g/L extract concentration. All of the three other extract combinations do not exhibit this suppressive effect as the 6.25 g/L, 12.5 g/L, 25 g/L extracts show almost no difference compared with control. However, all 50 g/L extracts show obvious growth hindrances. For *M. sativa*, the germination potential exhibited a larger difference than the germination rate under the same combination and extract concentration.

The extract of combination 2 (130.61 mg/L nitrogen and pH 8.5) exhibited a strong prohibitive result on the *M. sativa* radicle and hypocotyl growth. Fig. 3 also illustrated that the *M. sativa* radicle is more sensitive than the hypocotyl. When the extract concentration was 50 g/L, almost the growth of all radicals was obvious different compared to control, but the hypocotyls no significant difference.

### Effects of crude chicory leaf and root water-soluble extracts on germination and growth of *F. arundinacea*

**Figure 4:**
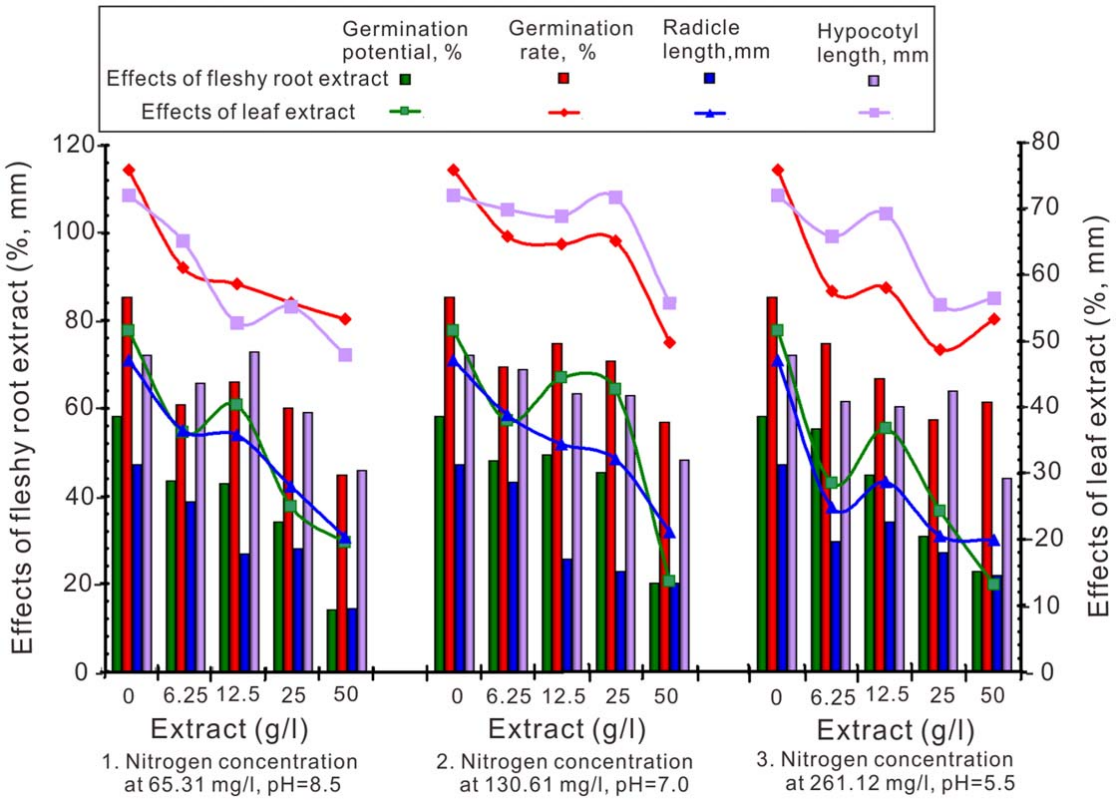
Effects of aquatic lixivium of different concentrations of chicory root and leaf extracts on the germination potential, germination rate, and radicle and hypocotyl length of *F. arundinacea* under varying nitrogen and pH treatments.

The results of *F. arundinacea* germination potential and rate, radicle and hypocotyl length with the crude chicory water-soluble extract concentrations under the three combinations of different pH levels and nitrogen concentrations are shown in Fig. 4. The integrative trends of germination potential, germination rate, lengths of radicle and hypocotyl were decreased when the extracts concentration increased from 0 to 50 g/l. The effect of the chicory root extracts of the three pH and nitrogen concentration combinations on the germination potential and rate of *F. arundinacea* was R_1_>R_2_>R_3_. When the root extract concentration was 50 g/L, all the trials were significantly different than the control. The extract concentration combinations 1 and 3 were different only up to 25 g/L with some change at 12.5 g/L. The effect of the chicory leaf extracts from the three pH levels and nitrogen concentration combinations on the germination potential and rate of *F. arundinacea* was R_3_>R_2_>R_1_. When the leaf extract concentration was 6.25 g/L, the treatments began to show a significant difference compared to the control. For *F. arundinacea*, all the R values for germination potential were greater than germination rate under the same treatment conditions.

Both root and leaf extracts at a concentration of 12.5 g/L, play a part in the growth of the radicle. However, extract concentrations of 25 g/L were needed to affect the lengths of the hypocotyl. Generally, *F. arundinacea* radicle and hypocotyl lengths showed obvious differences with increase of chicory root and leaf extract concentrations.

### Phenolics analysis and pH determination of chicory crude water-soluble extraction

Phenolics inhibit seed germination and seeding growth, which is generally accepted. Phenolics were analyzed using high-performance liquid chromatography (HPLC) (Fig. S1). The phenolics contents of chicory root and leaf extracts were determinated by caffeoylquinic acids and by caffeoylquinic acids and quercetin glucuronide, respectively. The root and leaf extracts made up 0.10 and 0.06 g per 100 g of dry mass, respectively. And pH of root and leaf extracts ranged from 4.88 to 5.84 and 4.34 to 6.57, respectively.

### Models analyses for allelopathic effects on equivalent coupling of nitrogen (X_1_) and pH (X_2_)

**Figure 5:**
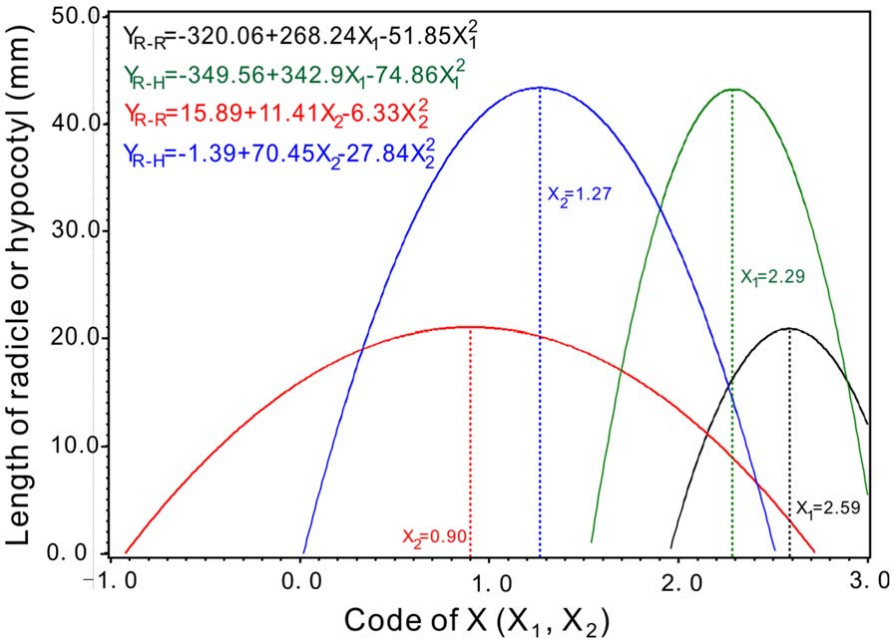
Nitrogen and pH equivalent coupling effects of models for the effects of aquatic lixivium of chicory root on the radicle and hypocotyl length of the three plants.

Under equivalent coupling effects of X_1_ and X_2_, curves of the quadratic models of radicles and hypocotyls lengths at aquatic lixivium of fleshy root were shown in Fig. 5. The lengths of radicles and hypocotyls had maximum values. This indicated that the coupling effects of X_1_ and X_2_ facilitated the growths of radicles and hypocotyls. Therefore, the responding X_1_ and X_2_ of maximum points compose optimal ranges of the seedling growth as 2.29<X_1_<2.59 and 0.90<X_2_<1.27 respectively. The ranges decoded as 149 mg/l<nitrogen (X_1_)<168 mg/l and 4.95<pH (X_2_)<7.0 (Table 2 and Fig. 5).

**Table 2:** Orthogonal matrix of the experimental design L_9_(3^4^).

TreatNo.	Nitrogen concentration(mg/l)		pH		Target plant
	matrix	code (X_1_)	matrix	code (X_2_)	matrix
1	1(65.31)	1	1(5.5)	1.00	2 (MS)
2	1	1	2(7.0)	1.27	1 (TR)
3	1	1	3(8.5)	1.55	3 (FA)
4	2(130.61)	2	1	1.00	1
5	2	2	2	1.27	3
6	2	2	3	1.55	2
7	3(261.12)	4	1	1.00	3
8	3	4	2	1.27	2
9	3	4	3	1.55	1

TR, MS and FA are stand for Trifolium repens, Medicago sativa and Festuca arundinacea respectively.

**Figure 6 pone-0031670-g006:**
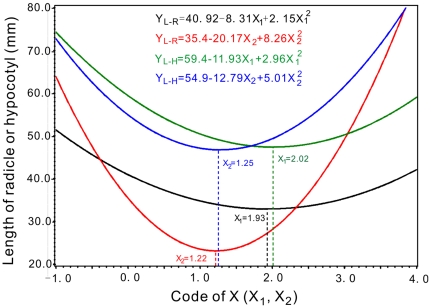
Nitrogen and pH equivalent coupling effects of models for the effects of aquatic lixivium of chicory leaf on the radicle and hypocotyl length of the three plants.

However, the curves of the quadratic models of radicles and hypocotyls lengths at aquatic lixivium of leaves were shown that the lengths of radicles and hypocotyls had minimum values on equivalent coupling effects of X_1_ and X_2_ (Fig. 6). It indicated that the coupling effects of X_1_ and X_2_ inhibited the growths of radicles and hypocotyls. Therefore, the responding X_1_ and X_2_ of minimum points compose ranges of the inhibitory effects as 1.93<X_1_<2.02 and 1.22<X_2_<1.25 respectively. The ranges decoded as 125 mg/l<nitrogen (X_1_)<131 mg/l and 6.71<pH (X_2_)<6.88 (Table 2 and Fig. 6).

**Figure 7 pone-0031670-g007:**
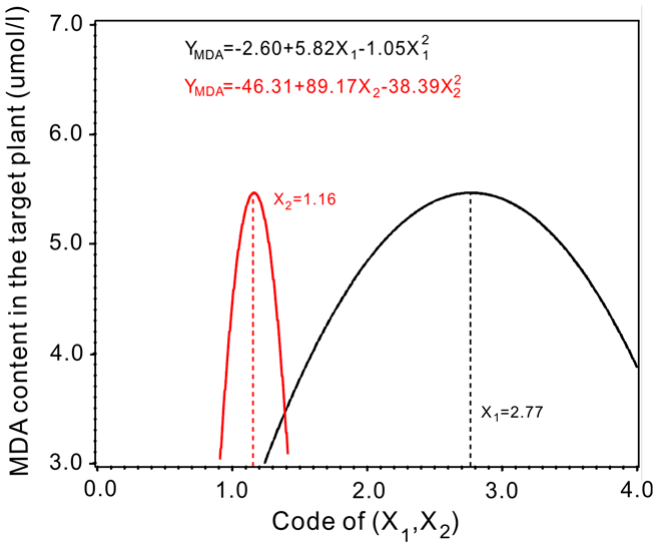
Nitrogen and pH equivalent coupling effects of models for the coupling effects in terms of the MDA content in the target plants.

Under equivalent coupling effects of X_1_ and X_2_, curves of the quadratic models of MDA content with X_1_ and X_2_ were shown in Fig. 7. The MDA contents had maximum values. The responding X_1_ and X_2_ of maximum points compose the point of the equivalent coupling effect at X_1_ = 2.77 and X_2_ = 1.16. The point decoded as nitrogen (X_1_) = 180 mg/l and pH (X_2_) = 6.38 (Table 2 and Fig. 7).

**Figure 8 pone-0031670-g008:**
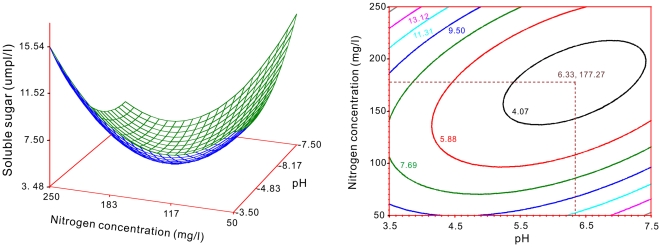
Response surface and contour chart for the coupling effects of nitrogen and pH on soluble sugar content in the target plants.

**Figure 9:**
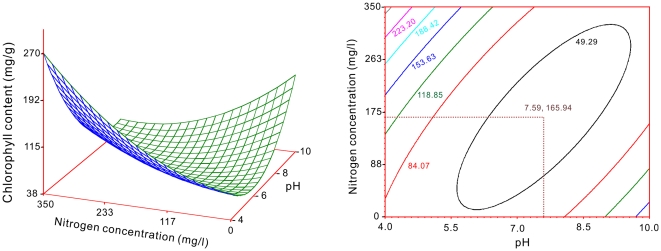
Response surface and contour chart for the coupling effects of nitrogen and pH on chlorophyll in content the target plants.

The contents of soluble sugar and chlorophyll in the target plant species as dependent variables with the nitrogen and pH treatments were respectively approached to two-variable quadratic regression models. The response surface and contour charts for the coupling effects of nitrogen and pH on the soluble sugar content shown that had a minimum point at 177 mg/l and pH = 6.33 (Fig. 8). Fig. 9 showed that on chlorophyll also had a minimum point at 166 mg/l and pH = 7.59.

## Discussion

The results of this research agree with that allelopathy is an accepted phenomenon of chemical interactions with widespread significance in natural ecosystems [9], [30]. The released allelochemicals naturally function on other plants, weeds or microorganisms in inhibitory or excitatory ways [31]. The occurrence of allelopathic interactions implies that plants are less passive than expected, as they interact directly with other plants by transmitting, receiving or responding to chemicals [32]. The allelopathic competence of the leaves and roots of many plants and trees are well documented in both laboratory and greenhouse experiments [31]. The results of this study accorded with above points to some extent (Fig. 1, 2, 3, 4).

Plants are affected by artificially controlled environmental factors, such as altering the media pH, increasing the soil nitrogen content and enhancing light. Plant culture pH would impact its growth by changing the decomposition of organic matter in the soil. It was reported that organic matter decomposition is greatest at a neutral pH and is reduced with an increase or decrease from neutral [33]; however, others have shown that organic matter decomposition increased from slightly acidic to alkaline pH [34]. Both of the viewpoints were inferred from Fig. 1 and Fig. 5, 6, 7, 8, 9 in this study. Inconsistent decomposition may be due to other complicated soil factors [35]. The culture pH also affects the enzymatic activity of the culture medium. For example, phenol oxidase and peroxidase were found to change with pH change. Therefore pH can affect normal plant growth through the alteration of the enzymatic activity. Additionally, nitrogen fertilization may have a profound impact on below-ground decomposition. It may modify the microbial community composition and thus alter the production of soil enzymes involved in the depolymerization of soil organic matter and plant litter [36]–[38]. In our experiments, the results that the points of maximum lengths of radicle and hypocotyls were at 149 to 168 mg/l range of nitrogen (Fig. 5). An increase in soil nitrogen availability may suppress the activity of oxidative enzymes. The effect of nitrogen on decomposition is dependent on the chemical composition of the organic matter [39] and may further affect plant growth metabolism.

Chicory root and leaf water-soluble extracts shown allelopathic effects as illustrated by the inhibition of germination potential, germination rate, and growth of the radicles and hypocotyls in all three target plants tested at different levels of extracts (Fig. 2, 3, 4). The most effective concentration was 50 g/L, which had a more pronounced inhibitory effect on the germination potential. The germination potential, germination rate, and the growth of the radicles and hypocotyls in three bioassay indexes decreased with increasing concentration of crude chicory root and leaf water-soluble extract. The seed germination was also strongly inhibited at the early stages of the experiment (Fig. 2, 3, 4). The roots may be the primary target as they are in direct contact with the leachates containing the chicory allelochemicals [40] and were particularly sensitive to allelochemicals [41]. The stem extracts were more inhibitory to radicle growth than were the root extracts for barley (*Hordeum vulgare L.*) [42]. In contrast, our experimental results in Fig. 2, 3, 4 demonstrated that leaf extracts have stronger inhibitory effects than the root extracts of chicory. Furthermore, in models analyses, there were maximum length of radicle and hypocotyls under aquatic lixivium extract of chicory root (Fig. 5). This inferred that the root extract facilitated the seedling growth; whereas minimum length of those under the extract of leaf (Fig. 6) inferred that the leaf extract inhibited the seedling growth. These may be caused by a higher concentration of phytotoxins in the leaves versus the roots. These findings accorded with the reports that the study of *Peganum harmala L.* demonstrated the inhibitory order as: leaves>stems>roots [8], that the aqueous extracts of lettuce leaf (*Lactuca sativa L.*) showed marked inhibition of seed germination of alfalfa [43] and similar results were also obtained when robinia (*Pseudo acacia L*) leaf was grown in soil mixed with barnyard grass, white clover, lettuce and Chinese cabbage at various concentrations [44].

The membrane fat peroxidation function of plants can increase in adverse conditions, which would increase the content of MDA as one of the decomposition products [1], [45]. Under equivalent coupling effects of nitrogen and pH, curves of the quadratic models showed a maximum MDA content in this study (Fig. 7). This inferred an inhibited effect at the points of the maximum MDA contents when the point was at X_1_ = 2.77 and X_2_ = 1.16. The point decoded as nitrogen (X_1_) = 180 mg/l and pH (X_2_) = 6.38 (Table 2 and Fig. 7). MDA concentration will increase when plants are exposed to diverse stresses or become senescent [45]. The higher MDA content subjected to a coupling effect of higher nitrogen (180 mg/l) and lower pH (6.38) environment in our experiment.

Soluble sugar is important in the osmotic regulation of substances within a plant body. In adverse conditions, an increase in the soluble sugar content is expected [1], [13]. In this study, the response surface and contour charts illustrated a minimum content of soluble sugar for the coupling effects of nitrogen and pH. The point of the minimum value was at 177 mg/l and pH = 6.33 (Fig. 8). This inferred that there was a stimulative effect at the point rather than an inhibitory effect.

Chloroplasts are the locations where photosynthesis takes place. Therefore, it is used to intercept light energy. The chlorophyll content directly impacts the photosynthetic ability of a plant, and to a certain extent, it reflects the level of leaf nitrogen [6], [46]. The result of this study showed a minimum value of chlorophyll content in the target plants when models' analyzing the coupling effects of nitrogen and pH (Fig. 9). This inferred that there was a strongest inhibitory effect at the point of the minimum value which was at 166 mg/l and pH = 7.59 (Fig. 9). Plants grown in nutrient solutions with a lower pH were negatively affected by the addition of HCl, which was added when the pH level was adjusted [46]. However, under acidic pH treatment (pH = 5.5, 261 mg/l nitrogen), the chlorophyll content of the target plants showed significant differences (Fig. 1 C). These results could have been caused by the presence of either excess protons or chloride ions in the solution [46].

**Figure 10:**
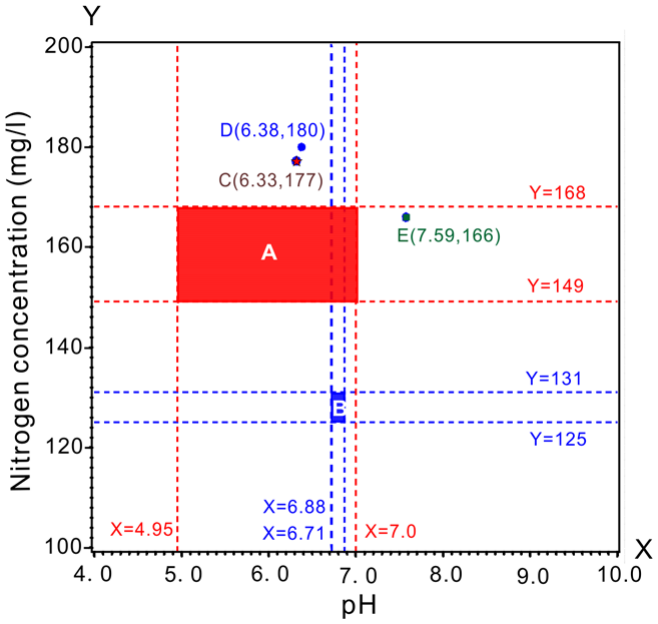
Distribution surface of nitrogen and pH for the effects on the seedling growth, soluble sugar, MDA and chlorophyll in the target plants.

Changing the soil pH and nitrogen composition can alter the secondary metabolism of allelochemicals. For investigating that the integrative effect of nitrogen and pH coupling via donor plant influence seedling growth of the target plant species, the results of models analyses (Fig. 5 to [Fig pone-0031670-g006] [Fig pone-0031670-g007] [Fig pone-0031670-g008] 9) were combined and plotted as distribution surface of nitrogen and pH (Fig. 10). Rectangular area ‘A’ and point ‘C’ indicated the results of Fig. 5 and 8 respectively. They were combined a location defined by 149 to 168 mg/l nitrogen supply and 4.95 to 7.0 pH level (area ‘A’) and at 177 mg/l nitrogen, 6.33 pH level (point ‘C’) (Fig. 10) because both of them had positive coupling effects on the target plants in this study. In contrast, the combinative treatments of the nitrogen and pH on chicory located at rectangular area ‘B’, point ‘D’ and ‘E’ (Fig. 10), which respectively indicated the results of Fig. 6, 7 and 9, had negative coupling effects on the target plant.

Phenolic compounds are often discussed in relation to allelopathy, which have been proven with phytotoxic perhaps due to their some minor fraction in many literatures [6], [40]. The total concentrations of phenolic acids in the ecotypes of important species were positively correlated with the total organic acid concentrations found in their respective soils [47]. Exposure to phenolic acids caused depolarization and an increased ion permeability in barley root membranes, and the inhibition of ion uptake was directly related to membrane perturbation and not to cytoplasmic changes [48]. Contents of phenolics for different treatments exist obvious difference, which basically increase with extractions concentrations rising.

Increasing the nitrogen level of cultivation can result in an increase in the conversion rate of organic to mineral nitrogen by soil microbes. Thus, a positive feedback mechanism is activated that leads to an increase the leaching of NO_3_^−^. The greater the total nitrogen uptake by plants, the greater the plant productivity [49], [50]. There were significant differences with regards to soluble sugar and MDA content among the 65, 130 and 261 mg/l treatment conditions (Fig. 1 A and Fig. 10). Similarly, a decrease in the soluble sugar concentration in the *Lycium barbarum* leaf was discovered with an increasing application rate of nitrogen fertilizer [51]. All of these changes were attributed to the complex physiological and biochemical mechanisms of plants. Therefore, these mechanisms need to be further investigated.

Different plants contain different genes. The diversity of genetic pool decides the diversity of various traits, so plants are expected to show differences in factors such as height, MDA content, chlorophyll content, etc. when they are grown in the same biotope. When the target plants were treated as factors, the soluble sugar, MDA and chlorophyll content showed significant differences. For example, *F. arundinacea* had a stronger nitrogen absorbability and assimilation even in the lower nitrogen supply and, as a result, had a better ability to maintain a high chlorophyll content [52]. Thus, these results can only be used to select for grass species with medium or poor nitrogen resistance.

### Conclusion

This study suggested a positive effect of nitrogen supply and pH level on allelochemical secretion from chicory plants. The nitrogen supply and pH level were located at rectangular area defined by 149 to 168 mg/l nitrogen supply combining 4.95 to 7.0 pH value and point located at nitrogen supply 177 mg/l, pH 6.33 when they were in equivalent coupling effects; whereas the inhibitory effects of equivalent coupling nitrogen supply and pH level were located at rectangular area defined by 125 to 131 mg/l nitrogen supply combining 6.71 to 6.88 pH value and two points respectively located at nitrogen supply 180 mg/l with pH 6.38 and nitrogen supply 166 mg/l with pH 7.59. Aqueous extracts of chicory fleshy roots and leaves accompanied by treatment at different soil pH values and nitrogen concentrations influenced germination, seedling growth, soluble sugar, MDA and chlorophyll of *F. arundinacea*, *T. repens* and *M. sativa*. Further research to quantify allelochemical activity as a function of pH and nitrogen supplementation and to investigate its consequence on hydrolytic enzyme activities may help to make precise decisions on adjusting the habitat of chicory.

## Materials and Methods

Two experiments composed this study. The experiment one was conducted in a completely automated PVC greenhouse, located at the Northwest A & F University (34°28′N, 108°07′E). The yearly average sunshine duration is 2,150 h. The average temperature is 12–14°C with the highest temperature between 39–40°C and the lowest temperature −21–−15°C, which represents the half-humid, warm temperate climate zone. The soil pH was 8.26, and the total nitrogen content in the top 20 cm of the soil was 0.00972% with a C∶N ratio of 11. The experiment two was germination test which was conducted in constant temperature culture base.

### Experimental materials

Our chicory (*C. intybus* L.) plants were transplanted from the first agricultural station in Northwest A & F University to the greenhouse as donor plants and placed 70 cm above the target plants. Our target species included *T. repens*, *F. arundinacea* and *M. sativa*, which were sown in pots as target plants.

### Experimental design

The experiment one was orthogonally designed to incorporate three factors at three levels using the L_9_(3^4^) orthogonal matrix. It contained three different target species (*F. arundinacea*, *T. repens* and *M. sativa*), three nitrogen treatments (X_1_: 0.5 N, 1 N and 2 N) and three pH treatments (X_2_: pH 5.5, pH 7 and pH 8.5). There were total of 9 treatments with three repetitions and comprised of 27 pots (Table 3).

**Table 3:** Design for the orthogonal L_9_ (3^4^) test.

Level	Factors		
	Target plant	pH	Nitrogen concentration
I	*Trifolium repens*	5.5	65.31 mg/L
II	*Medicago sativa*	7	130.61 mg/L
III	*Festuca arundinacea*	8.5	261.12 mg/L

Multiple 25 cm diameter pots were used as donor pots, which had a 10-cm diameter gap on the bottom. In this gap, a plastic funnel (12 cm diameter) was glued to the pot. The funnel was filled with pebbles and a wire netting (made of various meshes) was placed on the top to retain the sand placed in the pot. The funnel was connected to a polyethylene (PE) tube to allow the flow of leachate. Each of three pots was supplemented with and liquid nitrogen at appropriate pH every other day, which compensated for the water loss due to evaporation and leaching.

### Experiment one

#### pH and nitrogen treatment

To test the allelopathic effects of the chicory, we used sand with different pH levels and nitrogen concentrations in an orthogonal design of three factors with three levels (total of nine treatments). The pH values were adjusted to 5.5, 7, 8.5±0.02 using 0.001 mol/L NaOH and HCl. The concentrations of nitrogen treatments were 65.305, 130.610 and 261.122 mg/L, respectively (half, one and double times of the nitrogen content of modified Hoagland's nutrient solution).

#### Soluble sugar, MDA and total chlorophyll content analysis

Using the shoots (1 g fresh weight) corresponding to the 3 target plant species (*F. arundinacea*, *T. repens* and *M. sativa*) for each treatment, the amount of soluble sugar (sucrose), MDA and chlorophyll in 0.1 g was measured with a spectrophotometer (U-2001,Hitachi, Japan) at 450, 532 and 652 nm, respectively. Each experiment was conducted three times and results are presented in Fig. 1 as the mean of these three replicates.

### Experiment two

#### Crude water-soluble extraction of chicory for experiment two

When finished experiment one, the chicory plants in each donor pot were immediately washed with distilled water and divided into the above ground and the underground parts, then sliced up, air dried, ground into powder and passed through a 40 mesh sieve respectively. Eighteen (9×2) samples of the dry matter were got. Each one was soaked for 48 h (10.0 g of 100 mL distilled water) at 25°C with stirring once every 12 h. After a 10 minute centrifugation at 4000 rpm, the supernatant liquid was harvested and passed through filter funnels to obtain an original fluid with a concentration of 100 g/L. Each of the original fluid was then diluted to 6.25, 12.5, 25, 50 g/L and added with a control of distilled water for composing five levels of treatments. Totally 90 (9×2×5) samples of aquatic lixivium were got as treatments. The solutions were used to irrigate growing seeds of the target plants in Petri dishes every other day.

#### Allelochemicals analysis and pH determination of chicory crude water-soluble extraction

HPLC was applied to phenolics of chicory allelochemicals. A column of Diamonsil 18 C (2) (250 mm×4.6 mm ID) was placed. Linear gradient elution was carried out at a flow rate of 1 ml/min. Solvent A was 3% acetic acid in distilled water, and solvent B, acetonitrile with 3% acetic acid. Detected wavelength is 280 nm, and column temperature is 38°C. Identification and quantification of phenolic compounds were performed by comparing retention times, wavelength detection, and peak areas to those of standard compounds (Fig. S1). pH meter was used to determine pH of every extractions.

#### Germination test

The seeds of the three target plants species were disinfected with potassium permanganate for 15 minutes, and then rinsed repeatedly to remove the potassium permanganate completely with distilled water (5 to 6 washes). Next, the seeds were separately placed in 270 (90×3 repetition) Petri dishes (9 cm diameter and 1.7 cm deep). Fifty seeds were placed in each Petri dish. They were fitted with two pieces of 9 cm filter paper and moistened with 1 mL of the treatments respectively. The covered Petri dishes were incubated in completely dark conditions (Eyela, Eyelatron FLI-301NH, Japan) at 25°C for either 14 days for *F. arundinacea* or 10 days for *T. repens* and *M. sativa*. The percentage of germination potential (the 5th day for *F. arundinacea* and the 4th day for *T. repens* and *M. sativa*) and the germination rate (measured on the last experimental day) was recorded. The physiological characteristics of the radicles and hypocotyls and the length of both organs were also documented. This experiment was conducted three times and the results were presented in Fig. 2, 3, 4 as the mean of the three replicates.

### Data analysis and statistic methods

The germination potential, germination rate and radicle and hypocotyl length along with the sucrose, MDA and chlorophyll content of target plants were analysed using the analysis of variance (ANOVA) test. The five individual plants per target pot were averaged as an experimental unit. The target species and the treatments were treated as factors. Also, the fixed factors included three nitrogen concentrations and three pH levels. For the determination of significance, post hoc LSD tests were used to identify significantly different treatments.

For generic results, the factors nitrogen and pH were denoted by X_1_ and X_2_. The levels of X_1_ and X_2_ were coded (Table 2). The dependent variables, lengths of radicles and hypocotyls treated by root aquatic lixivium and leaves aquatic lixivium were denoted by Y_R-R_, Y_R-H_, Y_L-R_ and Y_L-H_ respectively. The MDA contents in the target species was denoted by Y_MDA_. These variables were approached and analyzed via two-variable (X_1_ and X_2_) quadratic regression models as [53]–[55]:
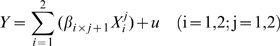
(1)Where, β is constants. For equivalent coupling effects of X_1_ and X_2_, thus,
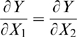
(2)Then, one-variable quadratic models of Y with X_1_ and X_2_ were respectively obtained and their quadratic curves were presented in Fig. 5, 6, 7.

Response surface and contour charts are respectively graphed for the soluble sugar and chlorophyll contents with their responding nitrogen and pH treatments (Fig. 8 and 9). Additionally, Distribution surface of nitrogen and pH for the results of two experiments was plotted in Fig. 10. The analyses and graphical procedures specified above were all performed using SAS (v8.2) [56].

## Supporting Information

Figure S1 **Chromatograms of chicory samples after water extraction; black, blue and green line respectively represent table sample, root extract and leaf extract.**
(DOC)
