# Supplementary material for: Models Analyses for Allelopathic Effects of Chicory at Equivalent Coupling of Nitrogen Supply and pH Level on F. arundinacea, T. repens and M. sativa
Source: PLoS One. 2012 Feb 22;7(2):e31670. doi: 10.1371/journal.pone.0031670 (PMC3285180; doi:10.1371/journal.pone.0031670)
Supplement: Figure S1 — Chromatograms of chicory samples after water extraction; black, blue and green line respectively represent table sample, root extract and leaf extract. (DOC) [file pone.0031670.s001.doc]

**PONE-D-11-16774**

**Models analyses for allelopathic effects of chicory at equivalent coupling of nitrogen supply and pH level on F. arundinacea, T. repens and M. sativa**

PLoS ONE

Supplementary material：

Figure S1. Chromatograms of chicory samples after water extraction; black, blue and green line respectively represent table sample, root extract and leaf extract.
